# Emerging Prevention and Treatment Strategies to Control COVID-19

**DOI:** 10.3390/pathogens9060501

**Published:** 2020-06-23

**Authors:** Vipul K. Singh, Abhishek Mishra, Shubhra Singh, Premranjan Kumar, Manisha Singh, Chinnaswamy Jagannath, Arshad Khan

**Affiliations:** 1 Department of Pathology and Genomic Medicine, Houston Methodist Research Institute, Houston, TX 77030, USA; amishra@houstonmethodist.org (A.M.); cjagannath@houstonmethodist.org (C.J.); 2 Department of Lymphoma/Myeloma, The University of Texas MD Anderson Cancer Center, Houston, TX 77030, USA; SSingh9@mdanderson.org (S.S.); MSingh4@mdanderson.org (M.S.); 3 Department of Medicine, Baylor College of Medicine, Houston, TX 77030, USA; premrank@bcm.edu

**Keywords:** COVID-19, SARS-CoV-2, drug repurposing, vaccine interventions, convalescent plasma, stem cell therapy

## Abstract

Severe acute respiratory syndrome coronavirus 2 (SARS-CoV-2), the causative agent of coronavirus disease 2019 (COVID-19), has now become a serious global threat after inflicting more than 8 million infections and 425,000 deaths in less than 6 months. Currently, no definitive treatment or prevention therapy exists for COVID-19. The unprecedented rise of this pandemic has rapidly fueled research efforts to discover and develop new vaccines and treatment strategies against this novel coronavirus. While hundreds of vaccines/therapeutics are still in the preclinical or early stage of clinical development, a few of them have shown promising results in controlling the infection. Here, in this review, we discuss the promising vaccines and treatment options for COVID-19, their challenges, and potential alternative strategies.

## 1. Introduction

The outbreak of coronavirus disease 2019 (COVID-19) has become a global health emergency on a pandemic scale. COVID-19 is a severe respiratory illness caused by the novel coronavirus severe acute respiratory syndrome coronavirus 2 (SARS-CoV-2) [1,2]. The emergence of SARS-CoV-2 was first detected in Wuhan, China, in December 2019. SARS-CoV-2 infections spread rapidly and globally, causing enormous distress and loss of life in no time. Though most SARS-CoV-2 infections cause only mild symptoms, 10–15% of patients develop the severe disease that requires hospitalization, with 5% requiring intensive care. In the United States alone, the Centers for Disease Control and Prevention reports over 2 million COVID-19 patients and over 110 thousand related deaths so far. Worldwide, SARS-CoV-2 has infected over 8 million people and caused over 400 thousand deaths as of 16 June 2020 (https://www.worldometers.info/coronavirus/).

**Figure 1:**
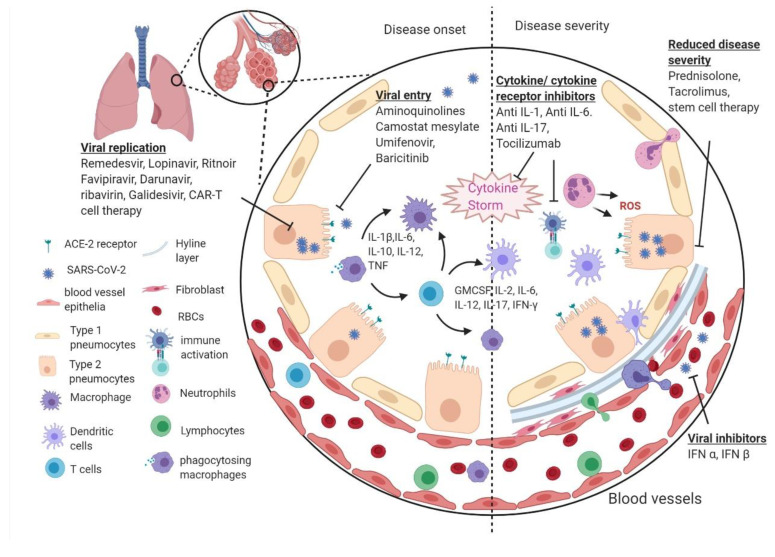
Pathogenesis of severe acute respiratory syndrome coronavirus 2 (SARS-CoV-2) infection: In the nasal cavity, the inhaled virus particles bind to angiotensin-converting enzyme 2 (ACE2) receptor of epithelial cells via its spike protein to gain intracellular entry and starts replicating [13]. The virus proliferates and simultaneously travels down the respiratory tract along the conducting airways, and clinical manifestation of symptoms start appearing [14]. In about 80% of the infected patients who develop only mild symptoms, the infection remains restricted to the upper respiratory airways only. However, in about 20% individuals, the infection migrates down to the lower respiratory tract and causes severe disease. The viruses reach the alveoli of the lungs and infect alveolar type II cells (type II pneumocytes) and propagate there [6,15]. Viral particles, after causing apoptosis of alveolar type II cells, work as a pulmonary toxin as they further infect type II cells in adjacent alveoli [8]. Subsequently, large areas of the lung will lose most of their type II cells, resulting in diffused alveolar damage with fibrin rich hyaline membranes, termed lung fibrosis. Other immune cells (macrophages, neutrophils, dendritic cells, T cells, etc.) now get recruited from the blood, and a vigorous innate and acquired immune response is initiated to reverse the caused damage which may lead to cytokine storm in some patients [16]. Cytokine storm is an uncontrolled over-production of cytokines (GM-CSF, IL-2, IL-6, IL-17, INF-g, etc.) that exacerbates the systemic inflammatory response and lung fibrosis, which could eventually lead to acute respiratory distress syndrome (ARDS).

As a newly emerged strain of coronavirus, SARS-CoV-2 was rapidly identified as the causative agent of the outbreak. SARS-CoV-2 contains a genomic sequence closely related to the 2003 severe acute respiratory syndrome (SARS) coronavirus (SARS-CoV) [3], and both these viruses are zoonotic. This is thus among some of the rare cases of an animal to human transmission of virus infection with pandemic potential [4]. Common symptoms of SARS-CoV-2 infection include fever, cough, and difficulty in breathing. In severe cases, the infection can result in death [5]. The pathogenesis of the SARS-CoV-2 infection is not completely clear yet. Our present understanding of COVID-19 pathogenesis so far is based on a limited number of investigated cases of SARS-CoV-2 and extrapolations from other similar coronavirus infections, such as SARS-CoV and MERS-CoV [6,7,8]. The probable courses of immunopathological events postulated based on limited studies on COVID-19 along with past studies with SARS-CoV have been illustrated in Figure 1. Transmission of infection between humans is facilitated by close contact with COVID-19 patients, as evidenced by the rapid global spread of the infection all around the world in a short period of time. SARS-CoV-2 likely originated in bats and may have amplified in an intermediate host before infecting humans. SARS-CoV-2 enters the human body via angiotensin-converting enzyme 2 (ACE2) receptors [9]. Current evidence indicates the mortality rate for SARS-CoV-2 could be approximately 3%, which is significantly lower than SARS-CoV (10%) and MERS-CoV (40%) [10]. Nonetheless, SARS-CoV-2 has much higher transmissibility (R_0_: 1.4–5.5) than both SARS-CoV (R_0_: 2–5) and MERS-CoV (R_0_: <1) [11].

Given the severity of COVID-19, the rapid global dispersion of SARS-CoV-2, and the declaration of the SARS-CoV-2 outbreak as a public health emergency of international concern by the World Health Organization, there is a pressing and increasing need for new diagnostics, vaccines, and therapeutic options for this new threat. However, in this early phase of the SARS-CoV-2 outbreak, a comprehensive prevention and treatment strategies is lacking due to limited knowledge about this virus. In efforts to mitigate the current COVID-19 pandemic, government agencies and scientific and medical communities worldwide are working on new approaches to reduce infection and decrease mortality rates among infected individuals [12]. So far, neither vaccines nor direct-acting antiviral drugs are available for the treatment of this disease. For now, most of the countries with a high case burden have adopted a combination of large-scale testing, contact tracing, isolation, and quarantine, in parallel with social-distancing measures to curb the COVID-19 pandemic, until a durable cure or prevention strategy is developed. While these measures have slowed down the spread of the disease and bought us more time to develop treatment and prevention strategies, there is a pressing need for a resilient solution that can effectively control the disease. The most effective strategy to control the disease is to develop a vaccine, and many industrial and academic organizations are racing to develop one but face many challenges to achieve this goal at an unprecedented timeline. Therapeutic drug options have been actively explored, including through many ongoing clinical trials, to reduce the severity of disease and death rate. This review provides an overview and analysis of most current advances in vaccines and therapeutics development against COVID-19 while also discussing some of the major roadblocks and challenges that we face to halt this emerging virus infection.

## 2. COVID-19 Drug Development

The drug development process involves a long series of safety assessments and clinical trials. Existing antiviral, antiparasitic, hypertension, and hypercholesterol drugs have demonstrated some success in preclinical assessments and clinical trials and may show utility if repurposed against COVID-19 (Table 1). The main objectives of repurposed therapeutic interventions are to reverse hypoxemia, provide adequate organ support, decrease viral load, and limit disease severity.

### 2.1. Pathogen Targeting Drugs

Remdesivir is a promising antiviral drug against RNA viruses, including SARS/MERS-CoV in cultured cells, mice, and nonhuman primate models [17]. Remdesivir is an adenosine analog that activates nucleoside triphosphate metabolites to inhibit viral RNA polymerases. In a nonhuman primate model, intravenous administration of remdesivir (10 mg/kg) resulted in 100% protection against Ebola virus infection [18]. Remdesivir is currently under clinical development for the treatment of Ebola virus infection [19]. In a recent double-blind, randomized, placebo-controlled trial of intravenous administration in adults hospitalized with COVID-19, remdesivir shortened the recovery time of patients by 31%, though it could not reduce the mortality rate to a significant extent. These initial results of remdesivir-mediated improvement of recovery time in clinical trials granted its FDA approval for critically ill COVID-19 patients [20,21,22]. Nonetheless, given high mortality despite the use of remdesivir, it is apparent that treatment with this antiviral drug alone may be less likely to be sufficient. A combination of remidesivir with other drugs is another alternative approach currently being explored in clinical trials to improve patient outcomes in COVID-19. Since a hyperactive immune response, leading to cytokine storm, has been found to be associated with increased disease severity and mortality, the addition of anti-inflammatory agent baricitinib to the remdesivir regimen is being explored in clinical trials (NCT04401579) to examine its potential in improving mortality outcomes. Several other existing antiviral drugs previously developed or used as treatments for other viral diseases are also being actively investigated as possible COVID-19 treatment options, with some already moved into clinical trials (Table 1). While the existence of evidence in support of these antiviral drugs is scant and preliminary in studies so far, more comprehensive studies on these drugs can build and extend the arsenal of anti-SARS-CoV-2 agents.

Chloroquine, a well-known drug used for the treatment of malaria and certain autoimmune disorders, has been previously shown to exhibit antiviral activity against HIV, Zika virus, and coronavirus [23,24,25]. Under in vitro culture condition, chloroquine blocks viral infection by increasing the endosomal pH required for virus/cell fusion [26]. It also prevents terminal glycosylation of the ACE2 receptor of the host cell, which is required for virus/cell fusion. Chloroquine mediated block of virus replication and entry in the host cell have been reported for many other viruses, including Borna disease virus, minute virus of mice, the avian leucosis virus, and hepatitis A virus [25,27]. These promising in vitro data suggested that chloroquine could be one of the promising drugs to treat COVID-19. However, the lack of robust preclinical data and potential safety issues of this drug remained to be addressed. To date, multiple clinical trials of chloroquine have been conducted either as a standalone drug or in combination with other drugs with and without randomization and placebo controls [28]. While no significant effect of Chloroquine on improvement in disease outcome could be ascertained in most of the studies, reports of adverse events have been observed in some cases including ventricular arrhythmias, QT prolongation, and other cardiac toxicity, indicating that this drug may pose risk to COVID-19 patients if not given methodically and at the right dose [29,30]. Interestingly, chloroquine is also known to affect immune system activity by mediating an anti-inflammatory response, which might reduce the pathology of COVID-19 due to the exaggerated inflammatory response it triggers during the progression of the disease [31]. However, no studies have yet evaluated the use of chloroquine for prophylaxis against COVID-19. Future studies that can better elucidate the most effective dose, schedule of administration, and adverse events along with its prophylaxis effect on the disease will confirm whether it has any potential in controlling COVID-19. 

The structure-based design of antiviral drug candidates 11a and 11b (Aldehyde based indole-2-carboxamide compounds), which target the SARS-CoV-2 main protease, recently exhibited in vitro antiviral activity and favorable pharmacokinetic properties in vivo, suggesting that they may also be promising drug candidates [32]. While the in vivo pharmacokinetics and toxicity studies showed encouraging results for these two new compounds, their efficacy against viral infection still needs to be validated in vivo in an appropriate experimental infection model, before forwarding to clinical trials. A more detailed review of the clinical studies of many other pathogen-targeting drugs, including remdesivir and chloroquine, has been described in other recent reports [21,33].

### 2.2. Host Directed Therapeutics

The high morbidity of SARS-CoV-2 patients is due to acute respiratory distress characterized by a cytokine storm that leads to increased plasma concentrations of proinflammatory cytokines IL-2, 7, 10, and 17; GM-CSF; interferon-γ-inducible protein 10; MCP-1; macrophage inflammatory protein-1 alpha; and TNF-α [34]. Tocilizumab, a monoclonal antibody that targets the interleukin-6 receptor, has demonstrated a favorable safety profile and promising clinical outcomes. Tocilizumab can suppress cytokine storms, improve respiratory function, and normalize body temperature [35]. However, this is a single observation study with a limited number of COVID-19 patients and hence may have a significant bias. COVID-19 mortality has been found to be associated with myocarditis in the setting of ARDS, and a TH17 type immune response has been reported to drive more severe viral myocarditis [36]. This suggests that anti-IL-17 therapy could be another potential approach in managing the disease severity and decreasing the mortality related to COVID-19-induced myocarditis.

Low-dose steroids such as prednisolone and tacrolimus have also been shown to inhibit pro-inflammatory cytokines that exacerbate lung pathology [37]. Given that pneumonia is secondary to a deleterious inflammatory process during COVID-19 disease progression, the use of prednisolone and/tacrolimus for severe COVID-19 lung injury patients thus could have a positive clinical effect. Clinical trials are already underway to evaluate the efficacy of these two immunosuppressive drugs in decreasing the secondary pathological manifestation of lung pneumonia in COVID-19 patients (NCT04341038). 

Another host-directed approach is to target the ACE/ACE receptor and renin-angiotensin-aldosterone system (RAAS) of the host cell to reduce the chances of infection. Hypertension drugs, lisinopril (ACE inhibitor), and losartan (angiotensin II receptor-blocker, ARBs) can increase the expression of ACE2 receptors, which may exacerbate viral load in cells [38]. However, some studies suggest that ACE inhibitors and ARBs could benefit patients with COVID-19 since ACE2 converts angiotensin II to angiotensin, which may have beneficial vasodilatory and anti-inflammatory properties. Though the usefulness of ACE inhibitors or ARBs is uncertain and based on retrospective cohort studies, ARB was recently approved for clinical trials to evaluate its potential for the treatment of COVID-19 patients [13,39]. Another alternative approach is to use recombinant human ACE2, as the virus may more readily bind to the soluble ACE2 in comparison to ACE2 receptors on the cell surface. This approach of preventing virus entry in the cells has shown promising preclinical results leading to its transition into clinical trials already [13,39]. Camostat mesilate is another potential candidate drug that is being considered as a host-directed therapy for COVID-19 patients. Camostat mesilate, which is used primarily for treating postoperative reflux esophagitis and for acute exacerbations of chronic pancreatitis, could block spread and pathogenesis of SARS-CoV in a pathogenic mouse model. It is known to inhibit the host serine protease TMPRSS2, which primes the spike protein of highly pathogenic human coronaviruses [40], and since SARS-CoV-2 is also dependent on host TMPRSS2 [13], it is a viable target for host-directed therapies. Based on more than 15 years of safe clinical track record in Japan and efficacy against SARS-CoV in preclinical studies, a double-blind randomized controlled clinical trial for repurposing this drug for COVID-19 is already in progress (NCT04353284).

## 3. Convalescent Plasma (CP) Therapy

Convalescent plasma (CP) is passive immunotherapy that has been used to improve the survival rate of patients with infectious diseases for over a century [55]. CP administration reduced mortality rates and shortened hospital stays in patients with SARS [56]. Various studies demonstrate that severely ill COVID-19 patients treated with CP showed a lower mortality rate compared to untreated controls [57]. A new study reported that critically ill COVID-19 patients (n = 10) treated with CP transfusion demonstrated significantly increased levels of neutralizing antibodies and depletion of viral load in 7 days. Clinical symptoms and paraclinical criteria in these patients improved within 3 days [58]. Placebo-controlled clinical trials to test CP therapy are ongoing in China (NCT04264858), and larger studies also started in the USA (Houston Methodist Hospital, Houston, Texas; Mount Sinai Medical Center, New York, New York, etc.), after it was approved by the US Food and Drug Administration for COVID-19 treatment [59,60]. CP from COVID-19-recovered patients is used to treat patients infected with SARS-CoV-2. The SARS-CoV-2-specific IgG antibodies received from SARS-CoV-2-recovered patients are passively transferred via transfused plasma and may neutralize viral particles and stimulate the complement system to encourage viral elimination [61]. While the results from some of the more recent clinical trials are encouraging [62,63], validation of these results from large randomized, placebo-controlled clinical trials is needed to further establish the effectiveness of CP therapy. CP dose and frequency are two of the criteria that may require more optimization to further improve the efficacy of CP therapy for COVID-19. Moreover, with regard to selecting the CP donor, the adequate titer of IgG and neutralization antibodies should be standardized and a robust donation program needs to be in place to make CP therapy a more viable treatment option for COVID-19. Early intervention, at the onset of symptoms, should also be considered as an important factor in enhancing the success rate of CP therapy for the treatment of COVID-19 patients. This is based on the observation that a better treatment outcome was achieved in SARS patients who were given CP before 14 days post infection, thus underlining the significance of early time administration of this therapy. Transfusion-related acute lung injury and infection enhancement that could occur due to subneutralizing concentrations of antibodies could be some of the major drawbacks of CP if these are not considered during the course of treatment. Relationship between SARS-CoV-2 RNA reduction and CP therapy, the optimal concentration of neutralizing antibodies required for effective treatment, and dynamic changes of different cytokine during treatment are some of the key parameters that, once defined, can further narrow the gap that exists for this therapeutic option. 

## 4. Stem Cell Therapy

Cell-based therapies, particularly stem cell therapies, have become a promising therapeutic approach to treat various diseases for which treatment has proven difficult. A few decades ago, researchers made the important observation that stem cells, including pluripotent/multipotent cells, were resistant to viral infection due to the expression of specific genes such as interferon-gamma-stimulated genes [64]. For example, there is direct evidence of stem cell protection against infection from Myxoma virus, a therapeutic oncolytic poxvirus. Hematopoietic stem cells can contain the Myxoma virus infection, while other cells, such as differentiated human monocytes, B-cells, and natural killer cells, cannot [65]. Recently, the USA, China, and several other countries have started stem cell therapy-based clinical trials to treat SARS-CoV-2 infection (Table 2). Even with the significant progress of stem cell-based approaches, limited cell sources, immunogenicity, and ethical issues remain some of the major limitations. Mesenchymal stromal cells (MSCs) have received attention due to their source potential, low invasive acquisition procedure, high proliferation rate, and lack of ethical concern. MSCs can be isolated from several tissues (e.g., adipose tissue, bone marrow, peripheral blood, cord blood, dental pulp, menstrual blood, Wharton jelly, buccal fat pad, and fetal liver) and can be stored for future treatment. They are easily expanded to clinical volume in a short amount of time, and their safety and efficacy have been thoroughly documented in many clinical trials [66,67,68]. While multiple clinical trials for the use of MSCs to treat various diseases are ongoing, so far none of them have been yet proposed for COVID-19 treatment.

**Figure 2:**
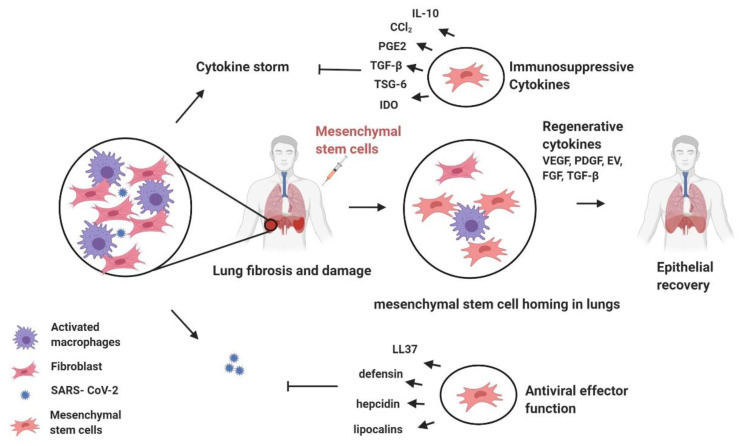
Schematic of Mesenchymal stem-cell-based therapy and its potential therapeutic action against COVID-19.

A 2016 study investigated the effect of MSCs therapy on influenza virus A/H5N1-infected mice and found that human MSCs reduced influenza virus A/H5N1-induced acute lung injury and increased overall survival [69]. Notably, during SARS-CoV-2 infections, the host immune system increases the production of inflammatory factors, inducing a cytokine storm that results in excessive production of immune cells and cytokines [34]. MSCs therapy may inhibit this release of proinflammatory cytokines and, owing to the regenerative properties of stem cells, they may also improve endogenous repair of injured tissues (Figure 2). MSCs secrete multiple growth factors (VEGF, PDFG, FGF, TGF-β, etc.) that regulate endothelial and epithelial permeability, suppress inflammation, and enhance tissue repair/regeneration [66]. Recent studies on stem cells’ ability to alleviate lung fibrosis has also been reported, which is a critical pathological factor in COVID-19 [70]. At the same time, MSCs are also known to exhibit strong antimicrobial effects through the secretion of antimicrobial peptides and proteins (LL-37, defensins, hepcidin, and lipocalins), which could benefit in reducing the viral loads as well during the progression of COVID-19 [71,72]. Thus, after the intravenous infusion of MSCs, a significant population of MSCs would naturally live in the lung, where they could cure COVID-19 via protecting alveolar epithelial cells, reclaiming the pulmonary microenvironment, preventing pulmonary fibrosis, and salvaging lung dysfunction. These hypotheses spurred the pursuit of MSCs therapy for SARS-CoV-2 patients. A recent study demonstrated that intravenous administration of clinical-grade human MSCs in seven severe COVID-19 patients resulted in improved functional outcomes with observable adverse effects [73]. The clinical benefit of MSCs should thus now be evaluated in a larger cohort of patients in the future. Moreover, the benefit of genetically engineered modified MSCs should also be assessed in COVID-19 patients. Genetically engineered modified MSCs have been used to treat several diseases, such as cancer, myocardial infarction, and acute liver failure. MSCs modifications include over-expression of cytokines, chemically engineered MSCs, physical preconditioning, or pharmacological preconditioning [74]. One important limitation of this approach is a dependency on stem cell banks for clinical-grade MSCs and limited speed of preparation.

## 5. Chimeric Antigen Receptor T-Cell (CAR-T) Therapy 

**Figure 3:**
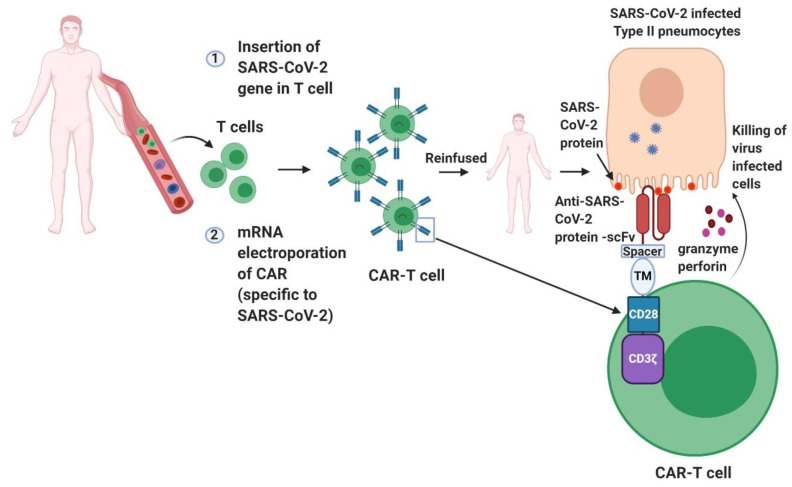
Engineering of chimeric antigen receptor T cells (CAR-T) targeting SARS-CoV-2. T cells can be engineered against SARS- CoV-2 by the expression of virus-specific receptor on their surface. After infusion into patients, CAR-T attach to the viral antigen present on the infected cells and become activated to release perforin and granzyme toxins that kill the infected cells. ScFv: Single-chain variable fragment; TM: transmembrane protein.

Scientific teams have now also begun investigating the prospects of CAR-T-based immunotherapy for treating COVID-19. CAR-T therapy represents an incredibly promising approach to reprograming T cells by expressing a synthetic receptor directed against the specific antigen to eliminate the virus-infected cells or cancer cells to control the disease progression [75]. CAR-T therapy has already revolutionized cancer immunotherapy, especially for the treatment of B cell malignancies [76,77]. Apart from their treatment potential against B-cell malignancies, CAR-T therapies have also shown early encouraging results for chronic hepatitis B virus (HBV) infection, HIV, and HBV-related hepatocellular carcinoma in recent studies [78,79]. The success of CAR-T therapy against some of these viral infections accentuates their immense potential against other infectious diseases as well, since pathogen-specific T cells play a pivotal role in controlling infections. This therapy involves the extraction of T cells from the patient/healthy donor’s bloodstream, and, after harvesting and expanding the T cells, they are reprogrammed to express a chimeric antigen receptor (CAR) targeting virus-infected cells (Figure 3). While the therapy could be cost-prohibitive for treating most types of viral infections, it could be a viable option for COVID-19 since no other definitive treatment option is available for this disease yet. It has been indeed demonstrated that CAR-T cells can be redirected to target the coronavirus responsible for the severe acute respiratory syndrome (SARS) [80]. These engineered T cells for SARS-CoV-specific antigen exhibited a functional profile comparable to that of SARS-specific memory CD8 T cells recovered from SARS-CoV-infected patients. This initial evidence suggested that CAR-T cells could thus be pursued for treatment for SARS-CoV-2. Nonetheless, the adoption of CAR-T therapy for clinical application against COVID-19 will require managing certain technical obstacles. One of the caveats is that CAR-T cells that stably express pathogen-specific T cell receptors could pose a risk of its proliferation and mediated excessive killing of infected cells, which could result in excessive inflammation and tissue injury. To overcome this problem, engineering of CAR-T cells could be done through mRNA electroporation, rather than using viral transduction, to obtain virus-specific T cells that will survive only for a limited time (Figure 3). The use of mRNA electroporation has been shown to result in only transient expression (3–5 days) of the CAR and thus is expected to decrease the risk for its off-target effects [81]. This technological adaptation can also reduce the viral contamination that can come from the viral transduction method of conventional CAR-T cell engineering. Transient CAR-T cells could also overcome the common cytotoxicity due to cytokine storm that has been observed after conventional CAR-T therapy. Cytokine storm has been one of the pathological manifestations observed in severely ill COVID-19 patients, which leads to increased lung inflammation. If lymphocytes are engineered using the transient mRNA transfection approach that keeps them active for a limited amount of time, this will likely minimize the potential of cytokine-storm-mediated cytotoxic effects. In this context, the dosing regimen and timing of administration of CAR-T cells should also be carefully assessed to rule out its possible cytotoxic effect for COVID-19 treatment.

## 6. COVID-19 and Vaccine Development

Unarguably, the best long-term strategy to curb the scale of the humanitarian and economic impact of the COVID-19 pandemic is to develop an effective vaccine that can prevent the new infections and stop the transmission of the disease. The scientific community and the vaccine industry have responded urgently to this epidemic of SARS-CoV-2 to support the development of vaccines against COVID-19. The demand of the situation and the availability of multiple vaccine technology platforms has also accelerated the development of many candidate COVID-19 vaccines at unprecedented rapidity, and some of these candidates have already entered human clinical testing. The pipeline of vaccines against COVID-19 has already reached more than 100 confirmed active candidate vaccines, though most of them are at exploratory or preclinical stages. As of June 4, 2020, 10 candidate vaccines that have made it to clinical stage represent diverse platforms including mRNA, DNA, Adenoviral/lentiviral/bacterial vector-based vaccines, and inactivated SARS-CoV-2 [82]. DNA and mRNA-based platforms offer the advantage of flexibility in terms of antigen manipulation and manufacturing speed. DNA and mRNA vaccines allow changing the antigen coding gene sequence in order to address the frequent mutations in the antigenic epitopes [83]. By comparing the genetic similarity between SARS-CoV-2 and SARS-CoV, a set of SARS-CoV-derived B cell and T cell epitopes that exactly match SARS-CoV-2 have also been identified recently [84]. As no mutations have been observed in the identified epitopes among the available SARS-CoV-2 genetic sequences, immune targeting of these epitopes for future vaccine candidates can also counteract the issue of genetic variations of the target antigen. Currently, there are no licensed DNA/mRNA vaccines for use in humans, but a few vaccines developed on these platforms have demonstrated potent immunity against various infectious disease targets and cancer in experimental animal models [85]. While enhanced delivery technologies, such as electroporation and liposomes, have increased the efficacy of DNA vaccines in humans, a shift towards RNA vaccine is clearly seen in the recent past. Non-infectious, nonintegrating, natural degradation, egg, and cell-free safety are some of the major safety advantages for mRNA vaccines over vector-based or DNA vaccines [86]. MRNA vaccines have also been found to stimulate a much broader range of innate and adaptive immune responses, which is desired against COVID-19. Phase 2 clinical trials investigating an mRNA vaccine (SARS-CoV-2 mRNA-1273) developed by Moderna have begun, and phase 3 trials are scheduled to begin soon [87]. 

Adenoviral (Ad) vector vaccines also represent a promising modern vaccine platform, as a variety of Ad vector vaccines have reached human clinical trials for infectious disease and cancer [88]. Since first-generation Adenovirus platforms (Ad5) have the disadvantage of induction of adenovirus neutralizing antibodies, a second-generation adenovirus vaccine platform with four deletions enabling multiple homologous doses is being used to develop COVID-19 vaccine [89,90]. Specific targeting of an adenovirus to dendritic cells has been found to be very critical for the efficacy of the Ad vector-based vaccine [91]. The replication-deficient feature of Ad vector vaccines is its key biologic property, and they have had an excellent safety profile in clinical translation so far. Ad vector vaccines can also be easily grown to high titer in cell culture, thereby facilitating their manufacture, regulatory approval, and clinical translation in a rapid manner [92]. Apart from that, the Ad vector vaccine also allows for the insertion of multiple transgenes, including not only the antigen of interest but also other genes that can enhance the response to vaccination. CanSino Biologics and the Academy of Military Medical Sciences (China) have developed a nonreplicating adenovirus serotype 5 (Ad5) vector carrying the gene for the SARS-CoV-2 spike protein, and clinical trials are underway in Wuhan, China [87,93]. At the University of Oxford, UK, phase 1/2 clinical trials using a chimpanzee adenovirus vaccine (ChAdOx1) carrying the gene for the SARS-CoV-2 protein are underway [3].

Dendritic cells/antigen-presenting cells modified with the lentiviral vector, which expresses viral proteins and immune-modulatory genes, is also a new vaccine platform being evaluated in clinical trials currently for COVID-19 (NCT04276896). Under this platform, DCs/APCs carrying a synthetic minigene of the viral structural proteins and a polyprotein protease of SARS-CoV-2 within the lentiviral vector is administered together with antigen-specific cytotoxic T cells. While there are fewer preclinical data and public information available about this platform, Phase I and II clinical trials are already underway to evaluate the anti-COVID-19 efficacy of these to lentiviral-based cell vaccines, and we can expect to see the results of these trials soon.

Some studies also suggest that vaccines for other diseases may confer resistance to SARS-CoV-2. Two recent reports suggested a correlation between Bacille Calmette-Guérin (BCG; antituberculosis)-vaccinated countries and reduced mortality in COVID-19 patients [94,95]. BCG is known to provide nonspecific protective effects through boosting innate immunity to treat malignancies such as bladder cancer, melanoma, lymphoma, and leukemia. Specifically, BCG may augment the first line of immune defense (so-called “trained immunity”) to provide a better defense against bacterial, parasitic, and viral infection [96]. Clinical trials in the Netherlands led by Dr. Mihai G. Netea [97] (NCT04328441), and in Australia, led by Dr. Nigel Curtis (NCT04327206), investigating the protection of BCG vaccines against the SARS-CoV-2 virus are ongoing. Some of the recombinant BCG candidate vaccines that have shown a better protection profile in early preclinical and clinical studies could be other potential candidates to explore for COVID-19 [98,99,100].

Moreover, since the immunity of the BCG vaccine is not known to last more than 10–15 years, revaccination of adults with BCG or recombinant BCG strains is another viable approach to consider, especially in countries where BCG is given at birth. We have curated the list of other vaccine candidates against SARS-CoV-2 from multiple journal sources that have recently moved into clinical trials, as mentioned in Table 3 [3,11,93].

## 7. Concluding Remarks

In summary, drug repurposing, plasma and stem cell therapies, and vaccines are pharmaceutical interventions that are being actively explored to limit the transmission of SARS-CoV-2 infection. An early clinical priority is to contain the spread of the infection via diagnostics that detect SARS-CoV-2 infection rapidly and accurately. A vaccine or drug that prevents future infections is another early priority. There are few potential SARS-CoV-2 drug and vaccine candidates for clinical trials in the pipeline globally. However, all promising treatment strategies should be investigated for efficacy and safety moving forward.

## Figures and Tables

**Table 1:** List of clinical trials of drugs to treat SARS-CoV-2 patients.

Drugs	Known Targets/Active against Diseases	Trial	References
**ANTIVIRALS**			
Aminoquinolines (chloroquine or hydroxychloroquine)	Antiparasitic	Phase III clinical trial	[17,41]
Remdesivir	Antiviral	Phase III clinical trial	[17]
Lopinavir/ritonavir combination	Anti-HIV	Phase II preclinical, clinical trial	[42]
Favipiravir	Antiviral	Phase III clinical trial	[43]
Darunavir	Anti-HIV	Phase III clinical trial	[44]
Ribavirin	Antiviral	Phase III clinical trial	[45]
Azithromycin (in combination with hydroxychloroquine)	Antimalaria	Phase III clinical trial	[17]
Interferon alpha	Antiviral	Phase III clinical trial	[46]
Interferon beta	Antiviral	Phase III clinical trial	[46]
Nitazoxanide	Antiparasitic/antiviral	Phase II clinical trial	[47]
Galidesivir	antiviral (hepatitis/ebola)	Phase III clinical trial	[48]
11a and 11b	SARS-CoV-2 main protease inhibitor	Pre-clinical	[32]
**HOST-DIRECTED THERAPIES**			
Renin-angiotensin-aldosterone system (RAAS) inhibitors	RAAS inhibitors	In vivo preclinical trials	[49]
Recombinant human angiotensin-converting enzyme 2 (ACE2)	ACE/ACE receptor inhibitors	Phase II clinical trial	[13]
ACE/ACE receptor inhibitors	ACE/ACE receptor inhibitors	Retrospective cohort study	[39]
Camostat mesylate	Angiotensin II receptor-blocker ARB	Phase II clinical trial	[13]
Baricitinib	Janus kinases (JAK) inhibitor	Phase III clinical trial	[50]
Upadacitinib and filgotinib	Rheumatoid arthritis (Janus kinases (JAK) inhibitor)	Preclinical	[51]
Umifenovir/Arbidol	ACE2 (Anti-influenza)	Phase III clinical trial	[52]
**REDUCED IMMUNOPATHOLOGY**			
Interleukin-6 inhibitors	IL-6 monoclonal antibodies	Phase III clinical trial	[35]
Interleukin-1 inhibitors	IL-1 monoclonal antibodies	Phase III clinical trial	[35]
Tocilizumab	Interleukin 6 receptors (IL-6R)	Preclinical, good safety profile, Phase II clinical trial	[35]
Interleukin-17 inhibitors	IL-17 monoclonal antibodies	Phase III clinical trial	[53]
Prednisolone	Steroids	Phase II clinical trial	[54]
Tacrolimus	Steroids	Phase III clinical trial	[54]

**Table 2:** List of cell-based clinical trials to treat SARS-CoV-2 patients.

Clinical Trial Number	MSCs Source	References
NCT04252118	MSCs	https://clinicaltrials.gov
NCT04299152	MSCs	https://clinicaltrials.gov
NCT04269525	Umbilical cord-MSCs	https://clinicaltrials.gov
NCT04276987	MSC-derived exosomes	https://clinicaltrials.gov
NCT04273646	Umbilical cord-MSCs	https://clinicaltrials.gov
NCT04313322	Wharton’s Jelly derived-MSCs	https://clinicaltrials.gov
NCT04302519	Dental Pulp- MSCs	https://clinicaltrials.gov
NCT04315987	NestCell-MSCs	https://clinicaltrials.gov

**Table 3:** Curated list of Vaccine Production Platform and Target for SARS-CoV-2.

Companies/Universities	Target for Vaccine	Estimated Timeline
Inovio Pharmaceuticals, USA	DNA-based vaccine	Expected to start in the next few months for human testing.
CureVac, Germany	RNA-based vaccines	Human trials would start this summer and are capable of generating a million doses.
Novavax	Nanoparticle-based vaccines	Expected to start in 3 months for human testing.
Johnson & Johnson	Adenovirus-vectored technology used for Zika, Ebola and HIV vaccine candidates	Expected to launch in 1 year to the market.
University of Hong Kong	Modified nasal spray influenza vaccine	Expected to start in 1 year for human clinical trials.
GeoVax-BravoVax	Modified Vaccina-Ankara-Virus-Like Particles (MVA-VLP) vaccine platform	Not available.
Shanghai East Hospital (Tongji University)-Stermirna Therapeutics	mRNA-based vaccine	Expected to start in 2 months for clinical trials.
